# Health Degradation Monitoring and Early Fault Diagnosis of a Rolling Bearing Based on CEEMDAN and Improved MMSE

**DOI:** 10.3390/ma11061009

**Published:** 2018-06-14

**Authors:** Yong Lv, Rui Yuan, Tao Wang, Hewenxuan Li, Gangbing Song

**Affiliations:** 1 Key Laboratory of Metallurgical Equipment and Control Technology, Wuhan University of Science and Technology, Ministry of Education, Wuhan 430081, China; lvyong@wust.edu.cn (Y.L.); wangtao77@wust.edu.cn (T.W.); 2 Hubei Key Laboratory of Mechanical Transmission and Manufacturing Engineering, Wuhan University of Science and Technology, Wuhan 430081, China; 3 Department of Mechanical, Industrial and Systems Engineering, University of Rhode Island, Kingston, RI 02881, USA; hewenxuan_li@my.uri.edu; 4 Smart Material and Structure Laboratory, Department of Mechanical Engineering, University of Houston, Houston, TX 77204, USA; gsong@uh.edu

**Keywords:** health degradation monitoring, early fault diagnosis, CEEMDAN, improved MMSE, smoothed coarse graining process, rolling bearing

## Abstract

Rolling bearings play a crucial role in rotary machinery systems, and their operating state affects the entire mechanical system. In most cases, the fault of a rolling bearing can only be identified when it has developed to a certain degree. At that moment, there is already not much time for maintenance, and could cause serious damage to the entire mechanical system. This paper proposes a novel approach to health degradation monitoring and early fault diagnosis of rolling bearings based on a complete ensemble empirical mode decomposition with adaptive noise (CEEMDAN) and improved multivariate multiscale sample entropy (MMSE). The smoothed coarse graining process was proposed to improve the conventional MMSE. Numerical simulation results indicate that CEEMDAN can alleviate the mode mixing problem and enable accurate intrinsic mode functions (IMFs), and improved MMSE can reflect intrinsic dynamic characteristics of the rolling bearing more accurately. During application studies, rolling bearing signals are decomposed by CEEMDAN to obtain IMFs. Then improved MMSE values of effective IMFs are computed to accomplish health degradation monitoring of rolling bearings, aiming at identifying the early weak fault phase. Afterwards, CEEMDAN is performed to extract the fault characteristic frequency during the early weak fault phase. The experimental results indicate the proposed method can obtain a better performance than other techniques in objective analysis, which demonstrates the effectiveness of the proposed method in practical application. The theoretical derivations, numerical simulations, and application studies all confirmed that the proposed health degradation monitoring and early fault diagnosis approach is promising in the field of prognostic and fault diagnosis of rolling bearings.

## 1. Introduction

Rolling bearings are a crucial part of the mechanical system, and its operational state directly affects the normal operation of the entire system. When the failure of a rolling bearing occurs, the change of the dynamic characteristics can be reflected from collected vibration signals [1,2,3,4], however, in most cases, the early fault of the rolling bearing is difficult to identify during the initial phase of the fault. The dynamic characteristics generated by early fault are usually submerged in strong background noise, thus, the weak fault is difficult to extract, and the exact time when the fault starts to happen is difficult to identify [5,6,7,8]. In most cases, the fault of the rolling bearing can only be identified when it has developed to a certain degree. At that moment, there is already not much time for maintenance, and could cause serious damage to the whole mechanical system in a short time thereafter. Therefore, to find an effective way to identify early faults of rolling bearings and conduct fault diagnosis in advance of serious faults is critical [9]. The early weak fault phase, namely when the fault begins to occur initially, needs to be identified, then some technical means could be implemented to avoid a serious fault from happening, for instance, by replacing machine parts or conducting maintenance after shutting down the mechanical system. The health degradation monitoring of rolling bearings can spare a great deal of time for maintenance, avoid unnecessary losses, and reduce the risk of catastrophic consequences to great extents [10,11]. The early weak fault phase can be identified during health degradation monitoring processes. Afterwards, early fault diagnosis can detect the weak fault of the rolling bearing, and determine the fault type of the rolling bearing, thereby, the parts can be maintained or replaced in time [12]. Hence, the health degradation monitoring and early fault diagnosis is significant in the field of prognostic and fault diagnosis of rolling bearings.

Many nonlinear signal processing methods have been proposed and developed in recent years, such as wavelet packet decomposition (WPD) [13], short-time Fourier transform (STFT) [14], independent component analysis (ICA) [15], empirical mode decomposition (EMD) [16], and empirical wavelet transform (EWT) [17]. Among all of the above methods, EMD [18] was proposed to adaptively decompose a time series into several approximating stationary time series, which is called the intrinsic mode function (IMF). Compared to other signal processing methods in the field of mechanical fault diagnosis, EMD is an adaptive nonlinear and nonstationary signal processing method, and has no requirement of basic functions. However, EMD still has modal aliasing and end effect problems, and the ensemble empirical mode decomposition (EEMD) [19,20,21] was proposed to alleviate the mode aliasing problem by utilizing the property of frequency uniform distribution of Gaussian white noise. Then complementary ensemble empirical mode decomposition (CEEMD) [22] was proposed to improve EEMD by adding positive and negative Gaussian white noise. In this way, it can guarantee the same decomposition effect as EEMD, and also reduce the sequence reconstruction errors caused by added white noise. However, CEEMD still cannot solve the problem of different orders of obtained IMFs caused by adding different noise signals, which promotes the emergence of the complete ensemble empirical mode decomposition with adaptive noise (CEEMDAN) [23,24]. CEEMDAN is an important development of the EEMD method, and CEEMDAN can realize the approximate perfect reconstruction of the decomposed signal, while also avoiding the problem that different noise signals generate different orders of IMFs.

Entropy is a method of measuring the complexity of a time series. The approximate entropy [25] was proposed at the first beginning, and sample entropy (SE) [26] was proposed afterwards as an improvement method. SE analysis can reflect the single scale time series information. In the related traditional entropy-based analysis methods, such as permutation entropy [27,28], information entropy [29,30], and wavelet packet entropy [31], they can both measure the regularity and orderliness of the time series. The SE value decreases along with the decrease of disorder of the time series. The SE algorithm is a typical method proposed to measure the complexity and quantization of time series [26] and has been successfully applied to physiological time series analysis. The SE algorithm compares the data with itself, namely self-matching. Hence, the approximate entropy is the measure of new information of the generated time series, which makes the results occasionally contain false information. Compared with other nonlinear dynamic methods, such as the Lyapunov exponent [32] and fractal dimension [33], the SE can obtain a stable estimation value with less data, and tolerate a larger range of parameter values which need to be selected.

To analyze the complexity of time series on different time scales, multiscale sample entropy (MSE) [34] was proposed based on SE. In the MSE algorithm, the coarse graining process is adopted to obtain the multiscale time series, instead of the original single-scale time series. Then the obtained signal is analyzed at each scale. The obtained MSE values can better reflect the dynamic characteristic changes of the time series. MSE has been widely applied to nonlinear and nonstationary data analysis [35,36], and it only requires short data to gain stable entropy values and has better anti-noise ability. The MSE greatly enriches the meaning of SE. The greater the probability of generating new patterns, and the more complex the time series is, the larger the entropy value is. Hence, the value can be used as the judgmental index and characteristic parameter to characterize the complexity of fault signals. MSE has a good performance in analyzing scalar time series, but when it comes to multivariate time series, it can only calculate the data of each channel separately. It cannot reflect the correlations and relationships between multivariate data, which can reflect the dynamic characteristics of multivariate time series more clearly and accurately. Later multivariate multiscale sample entropy (MMSE) [37] was proposed as a follow up study of MSE, and it can compute the MSE of multivariate time series, and deal with different embedding dimensions, delay time, and range of data channels in a strict and unified manner. On account of the internal essence of SE that it can measure the regularity and complexity of dynamic systems, MMSE can be adopted to continuously detect the characteristic change of time series [38,39]. Hence, it can be used to monitor the health condition and performance degradation of rolling bearings.

This paper proposed a novel approach to health degradation monitoring and early fault diagnosis of rolling bearings based on CEEMDAN and improved MMSE. The conventional MMSE is improved by the smoothed coarse graining process. During the health degradation monitoring of rolling bearings, all signals continuously collected from the mechanical system can be decomposed by CEEMDAN to obtain IMFs, and MMSE values of effective IMFs are computed. By analyzing the MMSE values of all signals in the long-term, the operating condition of the mechanical systems can be monitored. The improved MMSE of IMFs obtained by CEEMDAN can amplify the dynamic change characteristics to solve the problem that an early weak fault is difficult to identify and extract. After identifying the early weak fault stage, the early fault diagnosis of the rolling bearing can be accomplished by CEEMDAN. CEEMDAN can remove the strong background noise of the early weak fault signal, while extracting the fault characteristic frequency accurately. The proposed approach mainly aims at conducting health degradation monitoring of rolling bearings in complex mechanical systems, to determine the early weak fault phase in the whole wear-out process of a rolling bearing, and, thus, conduct early fault diagnosis of rolling bearings.

The rest of this paper is organized as follows: Section 2 introduces the methodology of CEEMDAN and MMSE improved by the smoothed coarse graining process and the proposed novel health degradation monitoring and early fault diagnosis approach. Section 3 presents the numerical simulations of CEEMDAN and MMSE, including the comparison between CEEMDAN and EEMD, MMSE with smoothed coarse graining process, and conventional MMSE. Section 4 presents the application studies of the proposed method to run-to-failure fault rolling bearing signals, to verify its effectiveness and validity. Section 5 concludes the paper.

## 2. Methodology

### 2.1. Complete Ensemble Empirical Mode Decomposition with Adaptive Noise

EMD is an adaptive signal decomposition method for analyzing nonlinear and non-stationary signals [18]. EMD is analogous to wavelet analysis, while EMD can overcome the difficulty that wavelet decomposition requires a reasonable choice of wavelet basis functions. Its essence is to decompose the original signal in order of different fluctuations. A series of IMFs with different amplitudes are obtained. The IMF in the EMD method must satisfy: (1) The number of extreme points and the number of zero crossings must be equal or, at most, one difference; and (2) the upper envelope consists of the local maximum points, the lower envelope consists of the local minimum points, and the average values of the upper and lower envelops are all 0. The detailed procedures of EMD are below:

(1) Given a signal *x*, and calculate all the maximum and minimum values of *r_k_* (*k* = 0), here *r_k_* = *x*.
(2) Use the cubic spline to interpolate all maximum and minimum points of *r_k_* to obtain the upper and lower envelopes *e*_max_ and *e*_min_, respectively.
(3) Calculate the average of the upper and lower envelopes *m* = (*e*_max_ + *e*_min_)/2.
(4) Calculate the IMF by *r_k_* − *m* = *h_k_*_+1_, and decide if *h_k_*_+1_ satisfies the conditions of IMF, if not, repeat (2)–(3) to obtain the envelope average that satisfies the conditions.
(5) Separate *h_k_*_+1_ from *x* to get *r_k_*_+1_, then let *k* = *k* + 1, repeat steps (2)–(4) with regarding *r_k_* as the original time series.
  (1) $$r_{k+1}=x-\sum_{i=1}^{k}h_{i}$$
(6) Repeat the above steps until the residual that meets the stop condition is obtained.

**Figure 1:**
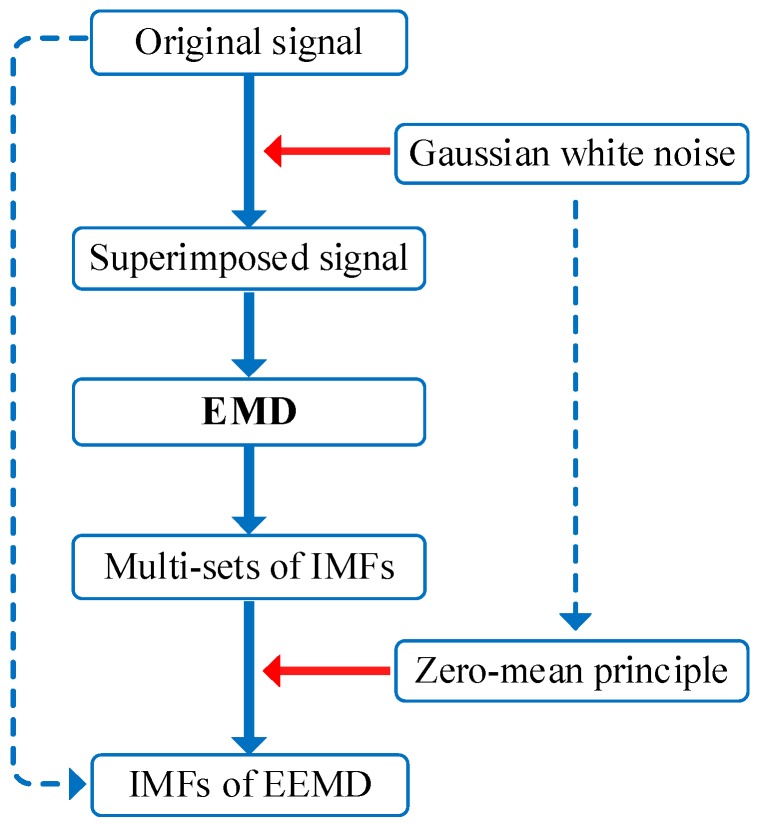
The illustration of EEMD.

To solve the mode mixing problem exists in EMD, which would result in IMF distortion, EEMD was proposed [19,20,21]. The EEMD algorithm is a noise-assisted signal processing method, and EEMD performs EMD multiple times on the signal superimposed with Gaussian white noise. The superimposed signal has continuity on various frequency scales, on account that Gaussian white noise has the statistical characteristics of uniform distribution. Hence, EEMD can help to alleviate the mode mixing problem in the IMF component. The computational framework of the EEMD is basically the same as EMD, and different white noise *w*^(*i*)^ (*i* = 1, …, *I*), where *I* is the ensemble size, can provide a consistent reference structure for the time domain distribution of the remaining components after each decomposition. The illustration of EEMD is shown in Figure 1, and the ensemble size determines the times of EMD conducted on the superimposed signal. The detailed procedures of EEMD are below:

(1) Add Gaussian white noise to the signal to form a superimposed signal *x*^(*i*)^ = *x* + *εw*^(*i*)^.
(2) Add Gaussian white noise to the signal to form a superimposed signal *x*^(*i*)^ = *x* + *εw*^(*i*)^.
(3) Perform EMD of *x*^(*i*)^ to obtain IMFs *d_k_*^(*i*)^ (*k* = 1, …, *K*), *K* is the number of all IMFs:
  (2) $$x^{\left(i\right)}=\sum_{k=1}^{K}d_{k}^{\left(i\right)}+r^{\left(i\right)}$$
(4) Adopt the zero-mean principle of Gaussian white noise to eliminate the influence by taking Gaussian white noise as a time domain distribution reference structure. Then the IMFs can be expressed as:
  (3) $${\bar{d}}_{k}=\frac{1}{I}\sum_{i=1}^{I}d_{k}^{\left(i\right)}$$

Among the above procedures, the input signal of each EMD is *r_k_*^(*i*)^ = *r_k_*_+1_^(*i*)^ − *d_k_*^(*i*)^, and there is no correlation between the *r_k_*^(*i*)^ of the EMD of different noise signals. It would make EEMD suffer from reconstruction error and generate different numbers of IMFs due to different EMD of various noise signals. To solve the reconstruction error in EEMD, CEEMD was proposed [22], in which Gaussian white noise is added into *x* in pairs (one positive and one negative) for twice EEMD, as shown in:

(4) $$\left[\begin{array}{c} y_{1}^{\left(i\right)}\\ y_{2}^{\left(i\right)} \end{array}\right]=\left[\begin{array}{cc} 1 & 1\\ 1 & -1 \end{array}\right]\left[\begin{array}{c} x\\ w^{\left(i\right)} \end{array}\right]$$

Such a method can reduce the reconstruction error to great extents, however, it still cannot guarantee the same number of IMFs generated by EMD of *y*_1_^(*i*)^ and *y*_2_^(*i*)^, which makes it difficult to compute the average. Thus, CEEMDAN [23,24] was put forward to ensure the same number of IMFs can be obtained, and reconstruction error can be eliminated at the same time. The first-order IMF obtained by CEEMDAN equals the first-order IMF obtained by EEMD, and the first residual *r*_1_ can be obtained by adding specific noise to make *r_k_* of each decomposition invariable.

The detailed procedures of CEEMDAN can be expressed as follows, among them *E_k_*(·) is defined as the operator of *k*th IMF, and *w*^(*i*)^ is defined as zero-mean Gaussian white noise:

(1) Perform EMD towards *x*^(*i*)^ = *x* + *β*_0_*w*^(*i*)^ (*i* = 1, …, *I*), and the first order IMF is:
  (5) $${\hat{d}}_{1}=\frac{1}{I}\sum_{i=1}^{I}d_{1}^{\left(i\right)}={\bar{d}}_{1}$$
(2) Compute the first residual: $r_{1}=x-{\hat{d}}_{1}$
(3) Perform EMD to obtain the first IMF of *r*_1_ + *β*_1_*E*_1_(*w*^(*i*)^) (*i* = 1, …, *I*), and the second IMF is:
  (6) $${\hat{d}}_{2}=\frac{1}{I}\sum_{i=1}^{I}E_{1}(r_{1}+\beta_{1}E_{1}(w^{\left(i\right)}))$$
(4) Compute *k*th residual for *k* = 2, 3, …, *K*:
  (7) $$r_{k}=r_{\left(k+1\right)}-{\hat{d}}_{k}$$
(5) Perform EMD to obtain the first IMF of *r_k_* + *β_k_E_k_*(*w*^(*i*)^) (*i* = 1, …, *I*), and the (*k* + 1)th IMF is:
  (8) $${\hat{d}}_{\left(k+1\right)}=\frac{1}{I}\sum_{i=1}^{I}E_{1}(r_{k}+\beta_{k}E_{k}(w^{\left(i\right)}))$$
(6) Return to step (4) to compute the next order IMF, and repeat steps (4)–(6) until the residual cannot be decomposed by EMD. The coefficient *β_k_* = ε*_k_* std(*r_k_*) allows the SNR to be selected during each iteration, and std(·) is the standard deviation operator.

### 2.2. Improved Multivariate Multiscale Sample Entropy

The SE [26] is an improved algorithm of approximate entropy, which can reflect the dissimilar probability of two similar sequences. The detailed procedures of SE are below:

(1) For the original time series ***X*** = {*x*_1_, *x*_2_, …, *x_N_*}, ***X***(*i*) = [*x_i_*, *x_i_*_+1_, …, *x_i_*_+*m*−1_], (1 ≤ *i* ≤ *N* − *m*) can be defined, *m* is the embedding dimension.
(2) Compute *d_ij_* (1≤ *j* ≤ *N* − *m*, *j* ≠ *i*) of ***X***(*i*) and ***X***(*j*), and calculate *num*(*d_ij_* < *r*) when *d_ij_* < *r*. *d_ij_* is the maximum absolute value of difference of ***X***(*i*) and ***X***(*j*). Define *B_im_*(*r*) = *num*(*d_ij_* < *r*)/(*N* − *m* − 1).
(3) Compute the mean value of *B_im_*(*r*), denoted by *B^m^*(*r*).
(4) As for the dimension of *m* + 1, repeat above procedures to obtain *B_im_*_+1_(*r*), then *B^m^*^+1^(*r*) can be obtained.
(5) The SE can be expressed as:
  (9) $$\mathrm{SE}(m,r,N)=\mathrm{In}B^{m}(r)-\mathrm{In}B^{m+1}(r)$$

**Figure 2:**
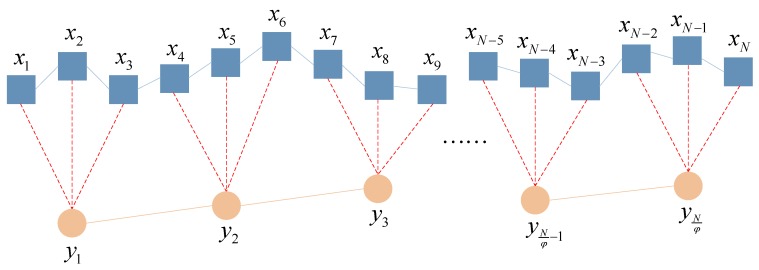
The illustration of the conventional coarse graining process.

Define the time scale on account of the coarse graining process aiming at the time series {*x_k_*_,*i*_}, in which *i* = 1, 2, …, *N*, and *k* = 1, 2, …, *p*. *p* is the number of variables, and *N* is the number of the points of each variable. As for any scale *φ*, the obtained multiple variable time series by the coarse graining process is as follows:

(10) $$y_{k,j}^{\varphi }=\frac{1}{\varphi }\sum_{i=(j-1)\varphi +1}^{j\varphi }x_{k,i}$$

where 1 ≤ *j* ≤ *N*/*φ*, *k* = 1, 2, …, *p*. Take scale = 3 as an example, the conventional coarse graining process is illustrated in Figure 2.

**Figure 3:**
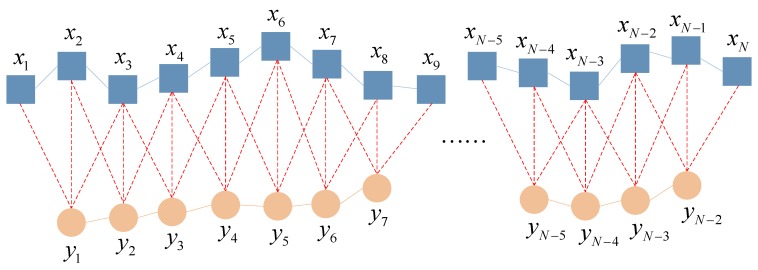
The illustration of proposed smoothed coarse graining process.

It can be seen from Figure 2 that the conventional coarse graining process compresses the time series by scale factors. Along with the increase of scale, the size of the coarse grained time series decreases, and when the length of the original time series is not an integral multiple of the scale factor, some of the data will be lost during the coarse graining process. All of the above phenomena would inevitably affect the calculation accuracy of the MMSE algorithm [37]. Aiming at improving such disadvantages, the smoothed coarse graining process, which adopts a sliding average method during the coarse graining process, is proposed. This method avoids data loss, and ensures the coarse-grained time series are the same length as the original time series at each scale, both significantly improving the accuracy of subsequent algorithms. The smoothed coarse graining process is illustrated in Figure 3.

Then the multivariate SE of each multiple variable *y_kj_^φ^* is computed, the multivariate embedded vector needs to be constructed in advance. Embedding theorem [40,41] is used to obtain the embedded vectors of the multivariate time series. For time series of *p*-variate time series, the multivariate embedding reconstruction is as follows:

(11) $$X_{m}(i)=[x_{1,i},\cdots x_{1,i+(m_{1}-1)\lambda_{1}},x_{2,i},\cdots x_{2,i+(m_{2}-1)\lambda_{2}},x_{p,i},\cdots x_{p,i+(m_{p}-1)\lambda_{p}}]$$

where ***M*** = [*m*_1_, *m*_2_, …, *m_p_*] ∈ *R^p^* is the embedding vector, and *λ* = [*λ*_1_, *λ*_2_, …, *λ_p_*] is the time delay vector. ***X****_m_*(*i*) ∈ *R^m^* (*m* = *m*_1_ + *m*_2_ + … *m_p_*).

As for the above multivariate time series, the MMSE can be computed as follows:

(1) Constitute multivariate embedding vector ***X****_m_*(*i*), and define the distance of any two vectors ***X****_m_*(*i*) and ***X****_m_*(*j*) as the maximum norm as follows:
  (12) $$D[X_{m}(i),X_{m}(j)]=\max_{l=1,2,\cdots ,m}\{|x(i+l-1)-x(j+l-1)|\}$$
(2) As for the composite delay vector ***X****_m_*(*i*) and a threshold *r*, determine the number of instances *P_i_*, where *D*[***X****_m_*(*i*), ***X****_m_*(*j*)] ≤ *r*, *j* ≠ *i*. Then compute the occurrence frequency *B_i_^m^*(*r*) = *P_i_*/(*N* − *n* − 1), where *n* = max{***M***} × max{*λ*}.
(3) Compute the average of *B*, denoted by *B^m^*(*r*).
  (13) $$B^{m}(r)=\frac{1}{N-n}\sum_{i=1}^{N-n}B_{i}^{m}(r)$$
(4) Extend the dimension of multivariate delay factor in (2) from *m* to (*m* + 1). Then, as for one embedding vector ***M*** = [*m*_1_, *m*_2_, …, *m_k_*…, *m_p_*], it can be converted into random space with the embedding vector of ***M*** = [*m*_1_, *m*_2_, …, *m_k_*_+1_…, *m_p_*] in *p* different ways. Thus, *p* × (*N* − *n*) vectors ***X****_m_*_+1_(*i*) can be obtained in *R^m^*^+1^, where ***X****_m_*_+1_(*i*) represents any embedding vector which increases embedding dimension *m_k_* to (*m_k_* + 1) for specific *k*. Due to the constant of the embedding dimension of other data channels in this process, the overall embedding dimension of multivariate time series increases from *m* to (*m* +1).
(5) Repeat procedures of (1)–(4) to compute all $B_{i}^{m_{k}+1}(r)$, and calculate the mean value *B_i_^m^*^+1^(*r*) upon *k*. Then compute the mean value *B^m^*^+1^(*r*) upon *i* in the (*m* + 1) dimensional space as:
  (14) $$B^{m+1}(r)=\frac{1}{p(N-n)}\sum_{i=1}^{p(N-n)}B_{i}^{m+1}(r)$$
  Here, *B^m^* (*r*) represents the similar possibility in *m* dimensional space of any two composite delay vectors, whereas *B^m^*^+1^ (*r*) represents the similar possibility upon (*m* + 1) dimensional space of two composite delay vectors.
(6) Then the MMSE can be expressed as:
  (15) $$\mathrm{MMSE}(M,\lambda ,r,N)=\mathrm{In}B^{m}(r)-\mathrm{In}B^{m+1}(r)$$

### 2.3. The Proposed Novel Health Degradation Monitoring Approach of Rolling Bearings

**Figure 4:**
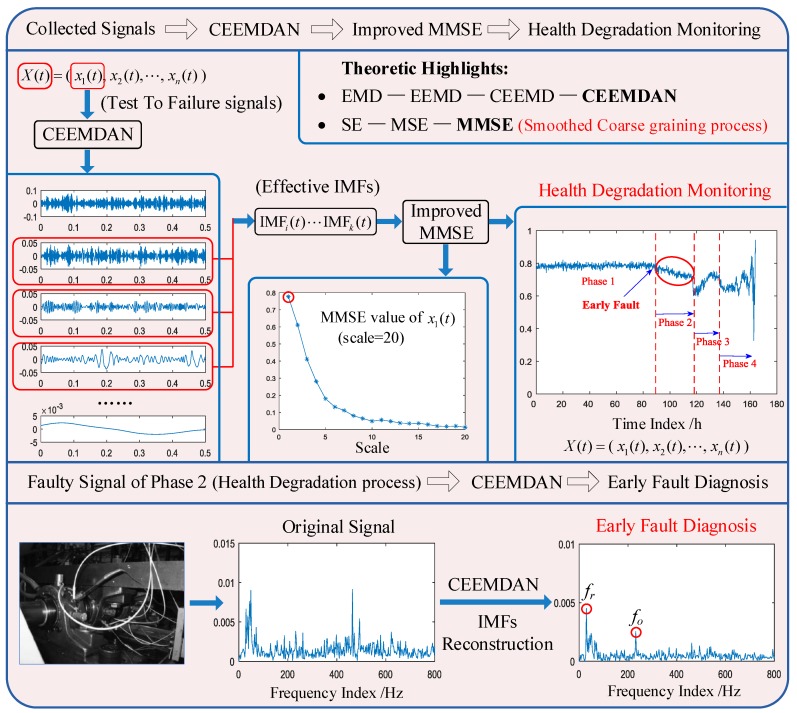
The proposed health degradation monitoring and early fault diagnosis scheme of a rolling bearing.

Studies on the IMFs of EMD and the derived methods demonstrate IMFs of EMD-derived methods can be adopted to accurately depict signal dynamics, and the unique properties of decoupling frequency information [42,43,44,45,46]. Hence, the intrinsic analysis method can be applied to detect the change regularity of complex dynamic systems. In this paper, a novel health degradation monitoring and early fault diagnosis approach is proposed based on CEEMDAN and improved MMSE, where the smoothed coarse graining process was proposed to improve the conventional MMSE. All continuously-collected signals of rolling bearings can be decomposed by CEEMDAN to obtain IMFs, and MMSE values of effective IMFs can be computed to accomplish health degradation monitoring of mechanical systems. After identifying the early weak fault phase, the fault frequency of rolling bearings can be extracted by CEEMDAN. The schematic diagram of the proposed method in this paper is illustrated in Figure 4.

## 3. Numerical Simulations

### 3.1. Simulation Research of Complete Ensemble Empirical Mode Decomposition with Adaptive Noise

**Figure 5:**
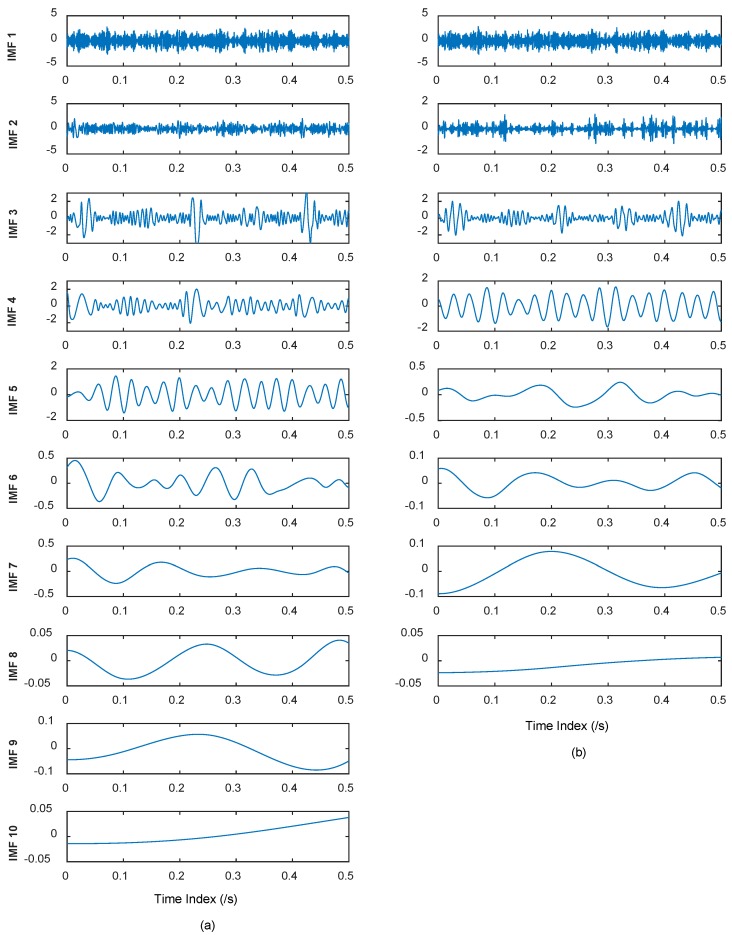
(**a**) Time domain plots of IMFs of EEMD; and (**b**) time domain plots of IMFs of CEEMDAN.

Mechanical operation signals usually have characteristics of frequency modulated signals, thus, the frequency modulated signals are used here to verify the effectiveness of the proposed method in tracking the characteristic change of the simulated signal. The vibrational signal of rolling bearing can be simplified to a signal model [47] as follows:

(16) $$x(t)=\alpha \sin(2\pi f_{b}t)[1+\beta \cos(2\pi f_{r}t)]$$

where *f_b_* denotes the characteristic frequency of the rolling bearing, and *f_r_* denotes the rotational frequency. *α* and *β* denote the power size. The collected rolling bearing signal usually has additive noise, and Gaussian white noise is adopted here to simulate the practical situation [48]. The filter bank property of EEMD and CEEMDAN is important in alleviating the current mode mixing problem, which is realized by taking advantage of the frequency uniformly-distributed property of Gaussian white noise. Hence, the simulated faulty rolling bearing signal can be simulated as follows:

(17) $$x_{1}(t)=\cos(2\pi f_{1}t)+\sin(2\pi f_{2}t)[1+\cos(2\pi f_{3}t)]+s$$

where *f*_1_ = 35 Hz, *f*_2_ = 80 Hz, *f*_3_ = 10 Hz. *s* denotes the Gaussian white noise added to the signal, with the variance of 2, and mean of 0. The sampling point is 2048, and the sampling frequency is 2048 Hz during the numerical simulation. The EEMD and CEEMDAN are applied to the simulated signal. The standard deviation of the added noise is 0.2, and the ensemble size is 50 in EEMD. Twenty-five pairs (positive and negative) of added noise are adopted in CEEMDAN. The time domain plots of IMFs of EEMD and CEEMDAN are shown in Figure 5.

**Figure 6:**
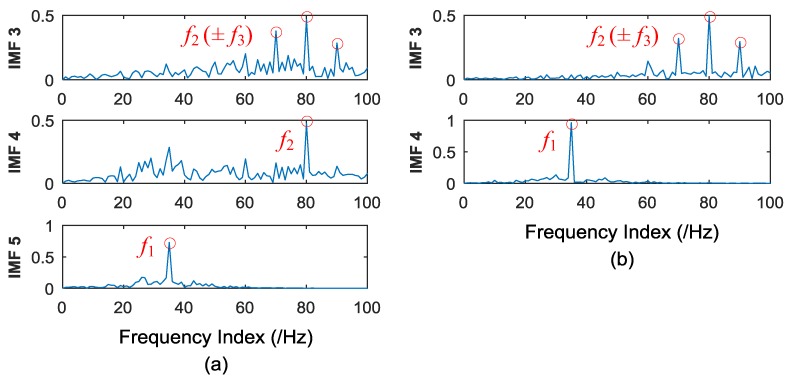
(**a**) Frequency domain plots of effective IMFs of EEMD; and (**b**) frequency domain plots of effective IMFs of CEEMDAN.

It can be seen from Figure 5 that 10 orders of IMFs are obtained by EEMD, while eight orders of IMFs are obtained by CEEMDAN. Obviously, CEEMDAN generates fewer orders of IMFs than EEMD does. To illustrate the effectiveness of CEEMDAN in alleviating the mode mixing problem, the correlation analysis is conducted to find the effective IMFs, and such a method was elaborated in authors’ previous studies [49,50]. During the correlation analysis, the 3rd, 4th, and 5th IMFs of EEMD, and the 3rd and 4th of CEEMDAN, are effective. The frequency domain plots of effective IMFs of EEMD and CEEMDAN are shown in Figure 6.

It can be seen from Figure 6 that *f*_1_ = 35 Hz can both be well extracted by EEMD and CEEMDAN with a slight difference. Furthermore, it can be observed from Figure 6a that the characteristic frequencies *f*_2_ (±*f*_3_), namely 80 Hz (±10 Hz), can be extracted in the 3rd IMF of EEMD, while *f*_2_ can also be found in the 4th IMF of EEMD. All characteristic frequencies are disturbed by some additive noise frequencies in the 3rd and 4th IMF of EEMD. This is exactly the mode mixing phenomenon existing in the EMD derived method. As for the performance of CEEMDAN, it can be observed from Figure 6b that characteristic frequencies *f*_2_ (±*f*_3_) can be extracted accurately in 3rd IMF of CEEMDAN, without additive noise disturbance. It indicates that there is no mode mixing problem appears in the obtained IMFs of CEEMDAN. It can be concluded based on the above analysis that CEEMDAN is superior to EEMD in alleviating the mode mixing problem, and can enable more accurate IMFs. Hence, CEEMDAN can be adopted in the proposed approach to health degradation monitoring and early fault diagnosis of rolling bearings.

### 3.2. Simulation Research of Improved Multivariate Multiscale Sample Entropy

**Figure 7:**
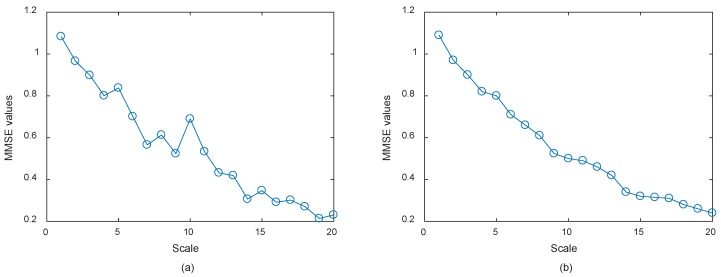
(**a**) MMSE values of the 1st second simulated trivariate signal adopting the conventional coarse graining process; and (**b**) MMSE values of the 1st second simulated trivariate signal adopting the smoothed coarse graining process.

To verify the effectiveness and superiority of improved MMSE with smoothed coarse graining process, here trivariate signal with additive Gaussian white noise is adopted for numerical simulation. The sampling point is 153,600 and the sampling frequency is 1024 Hz; namely, the sampling time is 150 s. The simulated trivariate rolling bearing signals are shown as follows:

(18) $$\begin{array}{l} x_{1}(t)=\cos(2\pi f_{1}t)[1+\sin(2\pi f_{2}t)]\\ x_{2}(t)=\sin(2\pi f_{3}t)[1+0.5\cos(2\pi f_{4}t)]\\ x_{3}(t)=\cos(2\pi f_{5}t) \end{array}$$

where *f*_1_ = 40 Hz, *f*_2_ = 15 Hz, *f*_3_ = 120 Hz, *f*_4_ = 35 Hz, *f*_5_ = 50 Hz. To simulate the fault characteristic change during the health degradation monitoring process, different amplitudes of characteristic signals containing noise are set at different periods. The time series can be divided into 150 subsequences 1024 points in length. The simulated trivariate faulty rolling bearing signal of multiple periods are given here as follows:

(19) $$X_{n}(t)=\{\begin{array}{c} 5x_{i}(t)+s,\;t=1∼50s\\ 10x_{i}(t)+s,\;t=51∼100s\\ 15x_{i}(t)+s,\;t=101∼150s \end{array},\;i=\;1,\;2,\;3,\;n=\;1,\;2,\;3.$$

where *s* denotes the Gaussian white noise with a variance of 1 and mean of 0. Then, the MMSE of the trivariate signal of different periods are computed to simulate the health degradation monitoring process. Here *m* = 5, *τ* = 1, sequence length *l* = 1024, scale *φ* = 20 were adopted in the simulation studies. The calculated MMSE results at different scales of the 1st second simulated trivariate signal are shown in Figure 7, respectively adopting the conventional coarse graining process and smoothed coarse graining process.

It can be seen from Figure 7a that the MMSE values of the time series monotonically decrease as a whole, but when the scales are 5, 8, 10, and 15, the MMSE values are greater than the former ones. This phenomenon indicates that MMSE values with the conventional coarse graining process have obvious fluctuations at certain scales, which could be caused by random noise, and would potentially lead to inaccurate and unstable results. While it can be seen from Figure 7b that the MMSE values of all scales monotonically decrease, it indicates MMSE values with the smoothed coarse graining process can reflect intrinsic dynamic characteristics of the faulty rolling bearing signals more accurately and steadily. Apart from that, it can also imply that the smoothed coarse graining process can alleviate the negative influence of random noise, which makes such a method more robust. Hence, the proposed improved MMSE can be adopted in the application studies for health degradation monitoring of rolling bearing.

**Figure 8:**
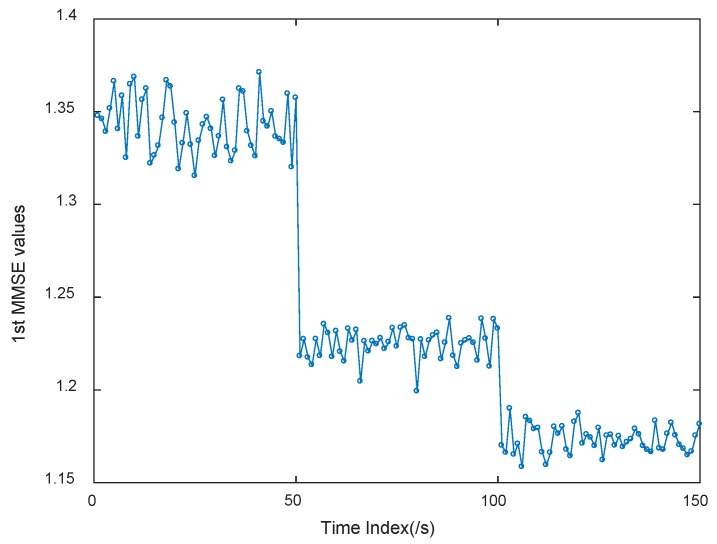
1st MMSE values of all subsequences of simulated trivariate faulty rolling bearing signal.

Based on the methodology and current studies of MMSE, if the MMSE values of a time series monotonically decrease, it means that this time series has low self-similarity, and only contains information at the smallest scale. Namely, the MMSE value of the smallest scale can be adopted as the indicator during health degradation monitoring of rolling bearings. Therefore, all the 1st (smallest scale) MMSE values of multiple periods of the simulated trivariate rolling bearing signal are computed, as shown in Figure 8.

It can be seen from the Figure 8 that the 1st (smallest scale) MMSE values can recognize the dynamic characteristic of the time series, when the characteristic signal grows larger, namely the simulated fault gets worse, the 1st (smallest scale) MMSE values indicate the heath degradation process. When the fault of rolling bearing happens or grows, the less complex the time series is, the less the MMSE value is. This means that MMSE values can be used for reflecting intrinsic dynamic characteristics of the faulty rolling bearing. Hence, the proposed approach by adopting MMSE can be used in health degradation monitoring of rolling bearings.

## 4. Application Studies on Health Degradation Monitoring and Early Fault Diagnosis of Rolling Bearings

### 4.1. Application Studies of the Proposed Health Degradation Monitoring Method of Rolling Bearings

**Figure 9:**
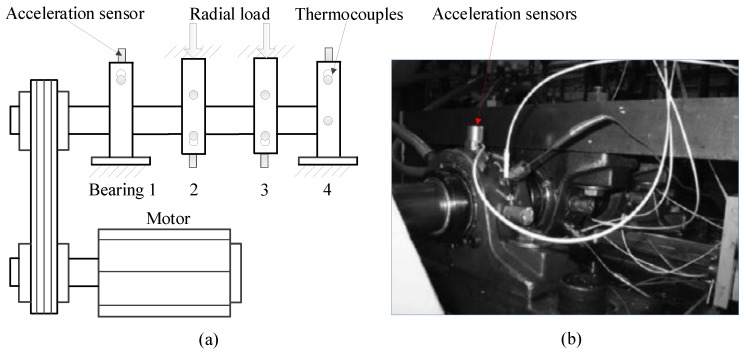
(**a**) The schematic diagram of the apparatus; and (**b**) a picture of the apparatus.

To verify the effectiveness of the proposed method in the application to rolling bearing signal processing, the run-to-failure fault rolling bearing signals provided by the Intelligent Maintenance Systems (IMS) Center of the University of Cincinnati (Cincinnati, OR, USA) are adopted for application studies. The recommended reference paper is given here [51]. The schematic diagram of the experimental apparatus and a photo of the apparatus is shown in Figure 9. There are four Rexnord ZA-2115 double-row bearings (Rexnord, Milwaukee, WI, USA) installed on the shaft. A PCB 353B33 high sensitivity quartz ICP accelerometer (PCB, Buffalo, NY, USA) was vertically installed on the bearing house. The position of the sensor placement is shown in Figure 9b. Then a test-to-failure experiment was conducted, and all data were collected by a NI 6062E DAQ card (National Instrument, Austin, TX, USA). The No. 2 dataset is used in this paper, during the experiment 984 sets of data files were obtained with a collection every 10 min (164 h in total), and each file contains 20,480 points at the sampling rate of 20 kHz. The rotational speed was set at 2000 r/min facilitated by an AC motor which was coupled to the shaft. Thus, the rotational frequency *f_r_* is 33.33 Hz. At the end of the experiment, bearing 1 had an outer ring wear-out failure, which indicated the experiment recorded the complete life test data.

As mentioned above, a run-to-failure experiment of a rolling bearing was conducted. The entire lifetime of the rolling bearing wear-out process can be described in four steps, denoted by Phases 1–4, as shown in Table 1. The self-balancing and self-healing process during Phase 3 is similar to the retardation effect during the crack growth of the beam, owing to the reason they have similar mechanical characteristics.

**Figure 10:**
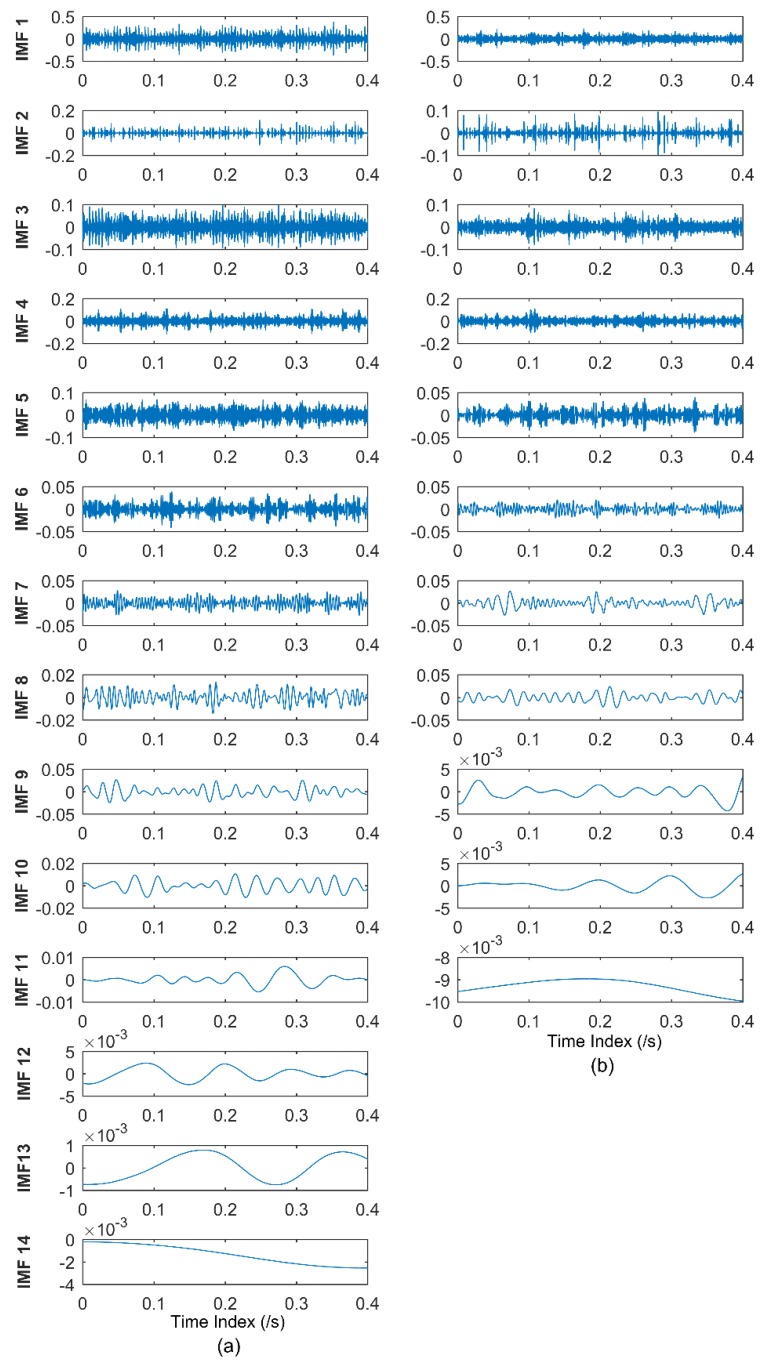
(**a**) IMFs obtained by EEMD; and (**b**) IMFs obtained by CEEMDAN.

To validate the effectiveness of the proposed health degradation monitoring and early fault diagnosis approach, EEMD and CEEMDAN of all vibration signals from 984 sets of data files are performed for comparison in subsequent analysis. Each data file has 20,480 points, and the sampling frequency is 20 KHz, as mentioned above. The sampling point is chosen as 8192 here, namely the first 8192 points are chosen for subsequent analysis out of 20,480 points. The EEMD and CEEMDAN of one set of data are shown in Figure 10. The standard deviation of the added noise is 0.2, and the ensemble size is 50 in EEMD, while 25 pairs (positive and negative) of added noise are adopted in CEEMDAN.

**Figure 11:**
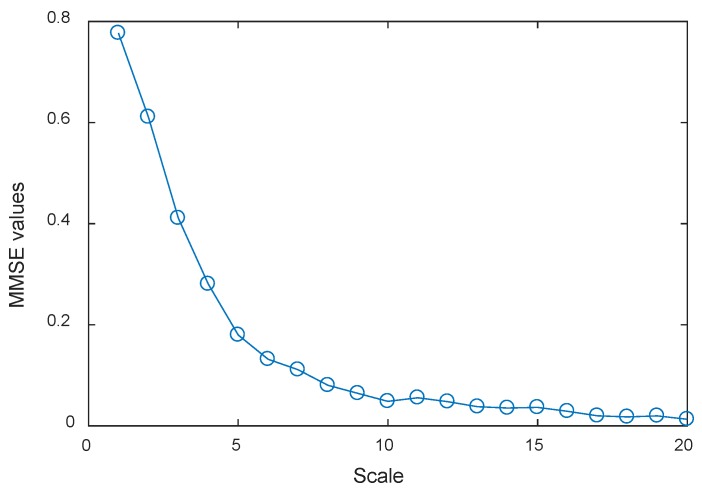
MMSE values of all scales via the proposed health degradation monitoring approach of the 100th set of data.

It can be seen from Figure 10 that 14 orders of IMFs are obtained by EEMD, while 11 orders of IMFs are obtained by CEEMDAN. Obviously CEEMDAN can generate fewer orders of IMFs than EMD, alleviating the mode mixing problem to obtain accurate IMFs denoting characteristic frequencies. In the process of extracting accurate frequencies, correlation analysis is adopted here. The correlation analysis has been studied in authors’ previous studies [49,50]. The noise component and the effective IMFs denoting fault features can be determined in the correlation analysis, to analyze the main component of the vibration signal to extract fault frequencies. During the correlation analysis, the top three effective IMFs of EEMD and CEEMDAN are adopted to obtain the MMSE values, and the same strategy is employed hereinafter. The MMSE values of the 100th set of data is given here as an example, as shown in Figure 11. Owing to the reason that MMSE values of a time series monotonically decrease, which has been illustrated in Section 3.2, the MMSE value of the smallest scale can be adopted as the indicator during the health degradation monitoring.

**Figure 12:**
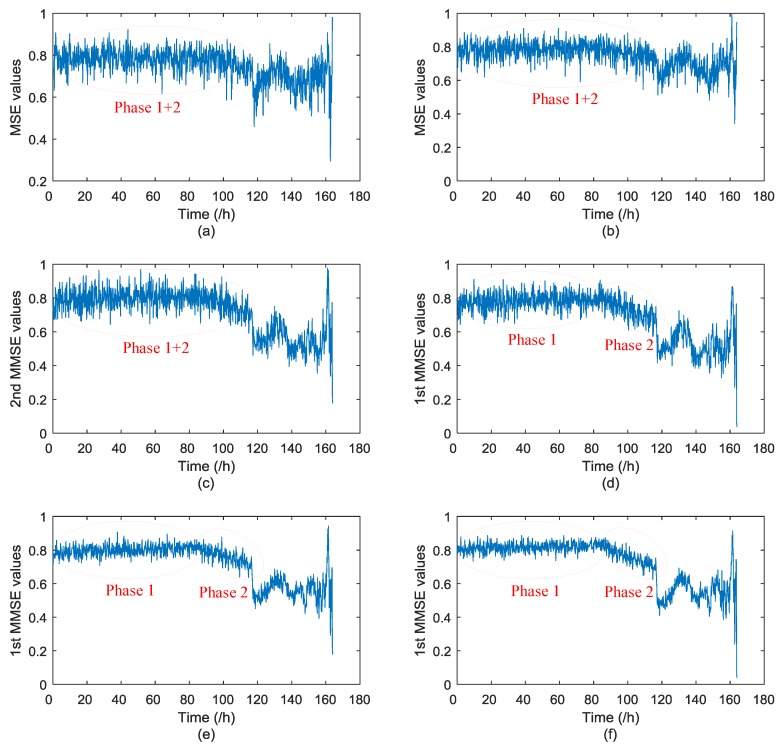
Comparative study of the six different methods. (**a**) The MSE values of the optimal IMF obtained by EEMD; (**b**) The MSE values of the optimal IMF obtained by CEEMDAN; (**c**) The 2nd MMSE values of effective IMFs obtained by EEMD; (**d**) The 1st MMSE values of effective IMFs obtained by EEMD; (**e**) The 1st MMSE values of effective IMFs obtained by CEEMDAN (conventional coarse graining process within MMSE); and (**f**) The 1st MMSE values of effective IMFs obtained by CEEMDAN (smoothed coarse graining process within MMSE).

**Figure 13:**
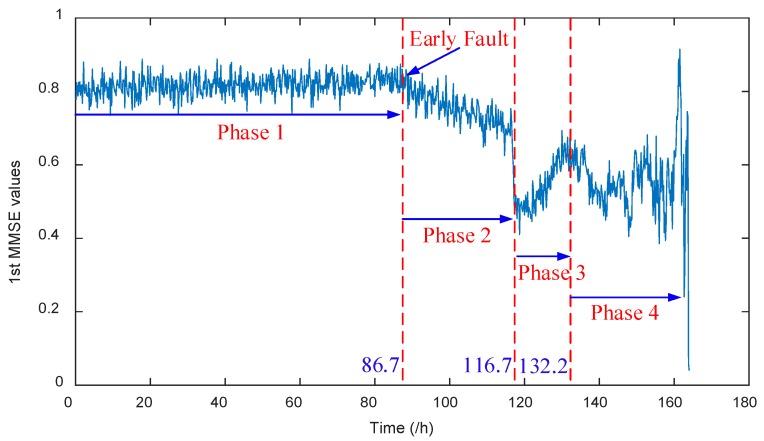
The 1st MMSE values of effective IMFs obtained by CEEMDAN (smoothed coarse graining process with MMSE).

To validate the superiority of CEEMDAN over EEMD, and the advantage of MMSE with the smoothed coarse graining process than the conventional one, six contrastive methods are shown in Figure 12 regarding the degradation data of the rolling bearing. Parameter values of *m* = 5, *τ* = 1, scale *φ* = 20, were selected for the algorithm. They are, respectively, the health degradation monitoring of the rolling bearing adopting MSE and EEMD, MSE and CEEMDAN, MMSE (2nd MMSE values) and EEMD, MMSE (1st MMSE values) and EEMD, MMSE with conventional coarse graining process and CEEMDAN, and MMSE with the smoothed coarse graining process and CEEMDAN. All six contrastive methods are shown as follows. To illustrate the effectiveness and superiority of the proposed method, the specific analysis of the proposed method, which is also presented in Figure 12f, is shown in Figure 13.

During the health degradation monitoring process, Phase 3 and Phase 4 are relatively easy to recognize since these are two phases when the fault, respectively, develops to the middle stage and deteriorates promptly. The proposed approach in this paper aims at early fault diagnosis after health degradation monitoring, which means Phase 3 and Phase 4 are not research priorities. Hence, only Phase 1 and Phase 2 are marked in the six contrastive methods in Figure 12 to compare the effectiveness of all methods, where Phase 2 denotes the early weak fault phase. It can be observed from Figure 12a–c that the health degradation monitoring method respectively adopting MSE values of the optimal IMF obtained by EEMD, MSE values of the optimal IMF obtained by CEEMDAN, and 2nd MMSE values of effective IMFs obtained by EEMD, cannot identify both Phase 1 and Phase 2 separately. It means these three methods fail to reveal the initial phase when an early fault happened; namely Phase 2 cannot be identified. The characteristic changes are more likely to be submerged by noise disturbances. From Figure 12d, it can be seen that the drop of the 1st MMSE values of effective IMFs obtained by EEMD can indicate Phase 2, the value change is more distinct than the former three methods but, still, the characteristic change is not obvious. From Figure 12e,f, it can be seen that health degradation monitoring based on CEEMDAN and MMSE (also improved MMSE) can both clearly show Phase 1 and Phase 2, respectively.

To illustrate the effectiveness of the proposed approach that integrates CEEMDAN and MMSE with smoothed coarse graining process, the enlarged results of Figure 12f are presented in Figure 13. Phases 1–4 are all marked in Figure 13 to show the whole health degradation monitoring process clearly. It can be clearly seen that the proposed approach in this paper can monitor the whole health degradation process distinctly, which can verify the effectiveness of the proposed approach. From Figure 13, it can be obtained that Phase 1 consist of 1–520 sets of data, and Phase 2 consists of 521–700 sets of data. Furthermore, to show the superiority of improved MMSE with smoothed coarse graining process over the conventional MMSE, the comparison between the results shown in Figure 12e,f based on numerical analysis are given in Table 2. The slopes of Phase 2 are obtained by computing the slopes of their approximate linear fitting curves.

It can be seen from the above numerical analysis of the proposed health degradation monitoring approaches, respectively adopting conventional and smoothed coarse graining processes, that the variances of Phase 1 and Phase 2 by using MMSE with adopting smoothed coarse graining are both smaller than adopting conventional coarse graining. This indicates that MMSE with a smoothed coarse graining process contributes to more stable and robust results during the health degradation monitoring process. The absolute value of the slope of Phase 2 by using MMSE by adopting smoothed coarse graining is greater than adopting conventional coarse graining. This indicates that the smooth coarse graining process can amplify the change characteristic during the health degradation monitoring process, which also contributes to obtaining more accurate results. Based on the above analysis, it can be seen that the proposed approach to the health degradation monitoring process adopting MMSE with smoothed coarse graining can obtain more stable and legible results than conventional MMSE, and can also obtain much better results than four other methods shown in Figure 12. In summary, the proposed approach in this paper shows good performance in revealing the fault change characteristics of rolling bearings in health degradation monitoring.

### 4.2. Application Research of the Proposed Early Fault Diagnosis Method of Rolling Bearings

It can be concluded from Section 4.1 that Phase 2 denotes the early weak fault stage, which is when the early weak fault happened for the very first time. Then, in this section, CEEMDAN is applied to the signal collected from the beginning of Phase 2, aiming at extracting the early weak fault characteristic frequency. The 520th set of data (denoting the signal around the 86.7th h) is the set of data that early weak fault started to happen at the very beginning during the wear-out process of the rolling bearing, and the fault characteristic frequency would not be obvious, or may have a very slight amplitude. The fault characteristic frequency would have greater amplitude along with the time. Hence, the 530th set of data (denoting the signal around the 88.3th h) is selected to extract the early weak fault. The sampling points for frequency spectrum analysis hereinafter is chosen as 8192. The detailed parameters of the faulty rolling bearing are shown in Table 3. The calculating method of the characteristic frequency and computed characteristic frequency are shown in Table 4.

**Figure 14:**
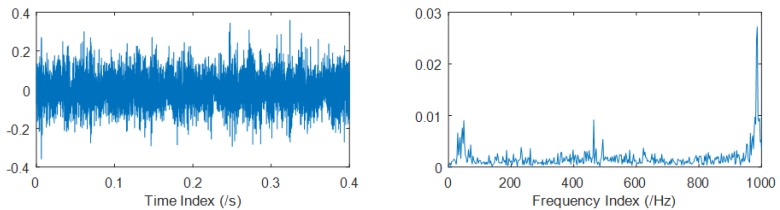
Time and frequency domain plots of the 530th set of data (denoting the signal around the 88.3th h), during the beginning of Phase 2 of the rolling bearing wear-out process.

The time and frequency domain plots of the selected data mentioned above are shown in Figure 14, as follows.

**Figure 15:**
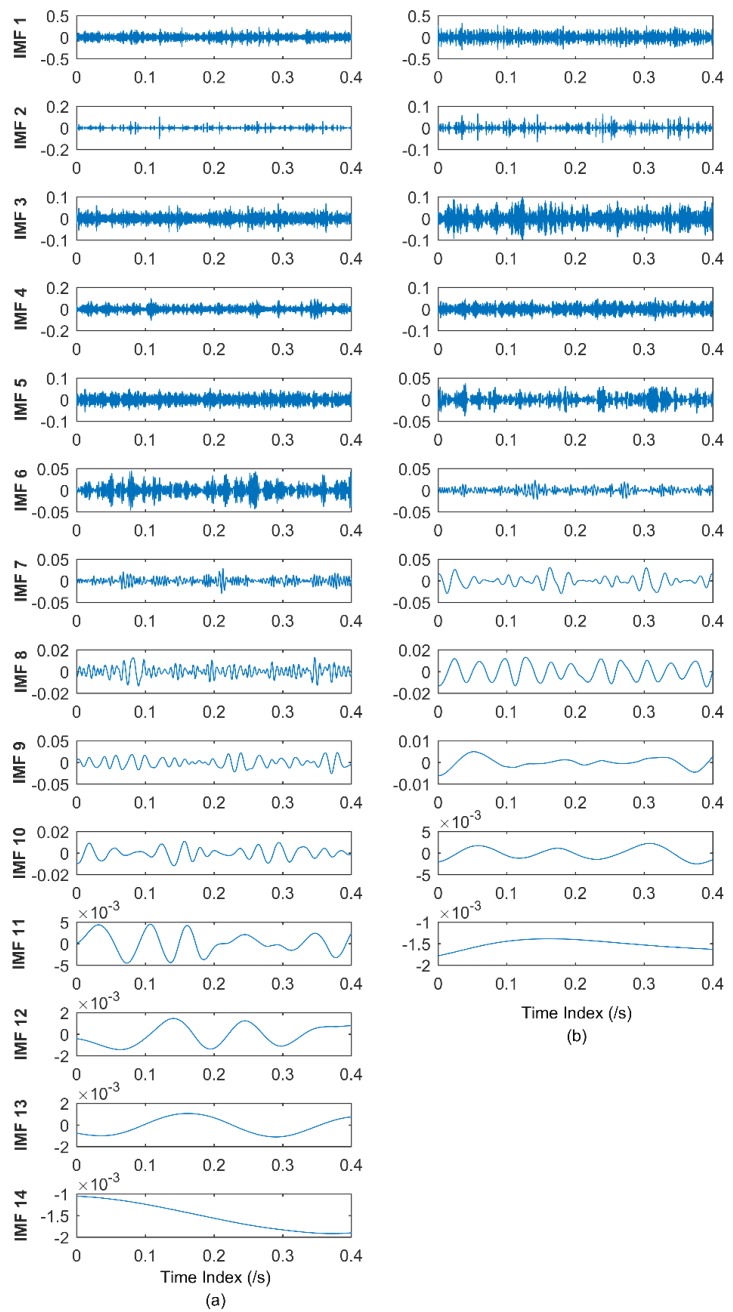
(**a**) IMFs obtained by EEMD; and (**b**) IMFs obtained by CEEMDAN.

EEMD and CEEMDAN are applied to the chosen data mentioned above. The standard deviation of the added noise is 0.2, and the ensemble size is 50 in EEMD, while 25 pairs (positive and negative) of added noise are adopted in CEEMDAN. The frequency domain plots of the optimal IMFs of EEMD and CEEMDAN are shown in Figure 15.

**Figure 16:**
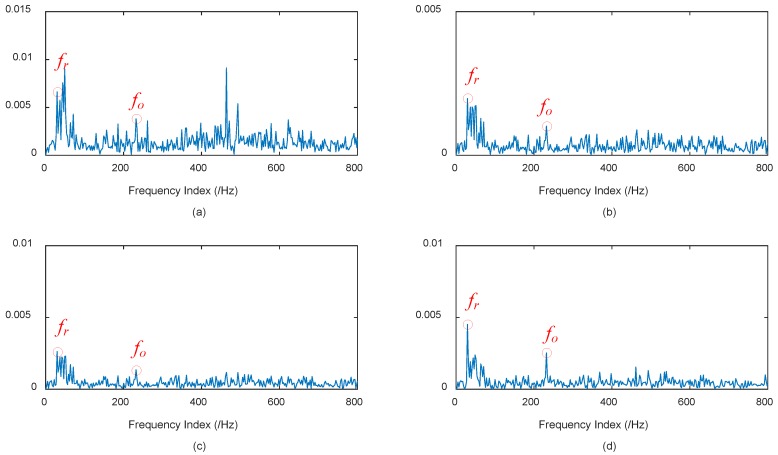
Frequency domain plots of the original signal and the reconstructed signals. (**a**) Frequency domain plot of the original early weak fault signal; (**b**) frequency domain plot of the reconstructed signal processed by WPD; (**c**) frequency domain plot of the reconstructed signal processed by EEMD; and (**d**) frequency domain plot of the reconstructed signal processed by CEEMDAN.

It can be seen from Figure 15 that 14 orders of IMFs are obtained by EEMD, and 11 orders of IMFs are obtained by CEEMDAN. The advantages of CEEMDAN have also been elaborated in Section 3.1 and the beginning of Section 4.1. The correlation analysis in the authors’ previous studies [49,50] is adopted to determine the effective IMFs, then IMFs denoting the noise components will be discarded during the IMF’s reconstruction. To illustrate the effectiveness of CEEMDAN, the frequency domain plot processed by WPD is also presented. The wavelet function db15 with 11 layers is adopted during WPD. During EEMD and CEEMDAN, the effective IMFs are selected to reconstruct the signal. The frequency domain plots of the original signal and the reconstructed signals respectively processed by WPD, EEMD, and CEEMDAN are shown in Figure 16.

It can be seen from Figure 16a that the original early weak fault frequency *f_o_* = 33.33 Hz is interfered by background noises to a great extent. There are great deal of redundant or disturbing frequencies, and fault characteristic frequency *f_o_* = 236.4 Hz is submerged in those frequencies. It can be seen from Figure 16b that, after WPD denoising, the fault characteristic frequency *f_o_* and rotational frequency *f_r_* can be found, but the results are far from good. Figure 16c shows after EEMD processing, the frequency domain plot has less disturbing frequencies, but the fault characteristic frequency *f_r_* cannot be well extracted, while in Figure 16d, after the proposed method in this paper, which is CEEMDAN processing, the fault characteristic frequency *f_o_* and rotational frequency *f_r_* can be well extracted. There are few redundant or disturbing frequencies, which indicates that CEEMDAN is superior to EEMD in alleviating the mode mixing problem, and can extract the accurate fault characteristic frequency. Hence, the early weak outer ring fault can be extracted by CEEMDAN, and the results can also ascertain the health degradation monitoring results hereinbefore. Based on the above analysis, it can be concluded that the proposed early fault diagnosis by adopting CEEMDAN can achieve a good result in application studies, and the proposed approach is promising in the field of fault diagnosis of rolling bearings.

## 5. Conclusions

In this paper, the research work elaborates the effectiveness of the proposed health degradation monitoring and early fault diagnosis of rolling bearings based on CEEMDAN and improved MMSE, which utilizes a smoothed coarse graining process. The theoretical derivation can demonstrate the significance of the novel approach in this paper, and the effectiveness is verified by numerical simulations and practical applications. The numerical simulation results indicate that CEEMDAN can alleviate the mode mixing problem and enable accurate IMFs, and improved MMSE can reflect intrinsic dynamic characteristics of rolling bearings more accurately and steadily. In the application research of the proposed health degradation monitoring method of rolling bearings, the results indicate that Phases 1–4 can be well distinguished by the proposed approach. The change characteristic can be reflected and the deterioration of the early weak fault phase can be amplified. It contributes a great deal to clearly identifying Phase 2 during the whole wear-out process, which is significant during the health degradation monitoring of rolling bearings. The superiority of improved MMSE with the adopted smoothed coarse graining process to conventional MMSE is also illustrated by numerical analysis. Afterwards, during the application research of the proposed early fault diagnosis method of rolling bearings, CEEMDAN can accurately extract the early weak fault characteristic frequency of the rolling bearing. The results of frequency spectrum analysis indicate that CEEMDAN is superior to EEMD in alleviating the mode mixing problem, and can enable more accurate IMFs to extract an early outer ring fault characteristic frequency *f_o_* and also the rotational frequency *f_r_*. The results of the early weak fault diagnosis can also ascertain the health degradation monitoring. Based on the analysis results of the simulated signal and practical experimental data, it can be concluded that the proposed health degradation monitoring and early fault diagnosis approach is promising in the field of prognostic and fault diagnosis of rolling bearings.

## Figures and Tables

**Table 1:** Four phases of the entire time of the rolling bearing wear-out process.

Phase 1	The bearing is operating normally, prior to the occurrence of the early weak fault.
Phase 2	The early weak fault occurs on the rolling bearing and interferes with its running condition slightly; this is the initial phase of early weak fault.
Phase 3	The fault develops into the middle stage, generating the self-balancing and self-healing phenomenon, also called the retardation effect. This is the phase that the rolling bearing fault tends to be serious.
Phase 4	The rolling bearing deteriorates promptly and has a serious fault. It usually results in the final breakdown of the rolling bearing.

**Table 2:** The comparison between health degradation monitoring methods adopting MMSE with a conventional coarse graining process and a smoothed coarse graining process.

Methods	Phase 1	Phase 2	
	Variance	Variance	Slope
MMSE (Conventional coarse graining)	0.78 × 10^−3^	2.21 × 10^−3^	−6.80 × 10^−4^
MMSE (Smoothed coarse graining)	0.34 × 10^−3^	1.16 × 10^−3^	−7.92 × 10^−4^

**Table 3:** Detailed parameters of faulty rolling bearing.

Detailed Parameters of Rexnord ZA-2115 Rolling Bearing			
Ball number *n*	Contact angle *α*	Ball diameter *d_r_*	Pitch diameter *D_w_*
16	15.17	0.331	2.815

**Table 4:** The calculating method and characteristic frequency of a Rexnord ZA-2115 rolling bearing.

Fault Type	Fault Frequency Computation	Fault Frequency
Outer ring fault	*f_o_* = 0.5*n*(1 − *d_r_*cos*α*/*D_w_*)*f_r_*	*f_o_* = 236.4
